# Anti-Heparanase Aptamers as Potential Diagnostic and Therapeutic Agents for Oral Cancer

**DOI:** 10.1371/journal.pone.0096846

**Published:** 2014-10-08

**Authors:** Suzanne C. Simmons, Hannaleena Jämsä, Dilson Silva, Celia M. Cortez, Edward A. McKenzie, Carolina C. Bitu, Sirpa Salo, Sini Nurmenniemi, Pia Nyberg, Juha Risteli, Carlos E. B. deAlmeida, Paul E. C. Brenchley, Tuula Salo, Sotiris Missailidis

**Affiliations:** 1 Department of Chemistry and Analytical Sciences, The Open University, Milton Keynes, United Kingdom; 2 Institute of Mathematics and Statistics, Rio de Janeiro State University, Rio de Janeiro, Brazil; 3 Manchester Institute of Biotechnology, University of Manchester, Manchester, United Kingdom; 4 Department of Diagnostics and Oral Medicine, Institute of Dentistry, University of Oulu, Oulu, Finland; 5 Medical Research Center and Oulu University Hospital, Oulu, Finland; 6 Institute of Diagnostics, Department of Clinical Chemistry, University of Oulu, Oulu, Finland; 7 Laboratório de Radiobiologia, Instituto de Radioproteção e Dosimetria, Rio de Janeiro, Brazil; 8 Renal Research Group, University of Manchester, Manchester, United Kingdom; 9 Graduate Program in Estomatopatologia, Piracicaba Dental School, University of Campinas, Piracicaba, São Paulo, Brazil; 10 Institute of Dentistry, University of Helsinki, Helsinki, Finland; 11 Institute of Biophysics Carlos Chagas Filho, Federal University of Rio de Janeiro, Rio de Janeiro, Brazil; King's College London, United Kingdom

## Abstract

Heparanase is an endoglycosidase enzyme present in activated leucocytes, mast cells, placental tissue, neutrophils and macrophages, and is involved in tumour metastasis and tissue invasion. It presents a potential target for cancer therapies and various molecules have been developed in an attempt to inhibit the enzymatic action of heparanase. In an attempt to develop a novel therapeutic with an associated diagnostic assay, we have previously described high affinity aptamers selected against heparanase. In this work, we demonstrated that these anti-heparanase aptamers are capable of inhibiting tissue invasion of tumour cells associated with oral cancer and verified that such inhibition is due to inhibition of the enzyme and not due to other potentially cytotoxic effects of the aptamers. Furthermore, we have identified a short 30 bases aptamer as a potential candidate for further studies, as this showed a higher ability to inhibit tissue invasion than its longer counterpart, as well as a reduced potential for complex formation with other non-specific serum proteins. Finally, the aptamer was found to be stable and therefore suitable for use in human models, as it showed no degradation in the presence of human serum, making it a potential candidate for both diagnostic and therapeutic use.

## Introduction

Heparanase is a β-1,4-endoglycosidase enzyme [1] that participates in extracellular matrix (ECM) degradation and remodeling [1]. The heparanase gene was first cloned in 1999 by the Vlodavsky and Parish groups in the seminal back to back Nature medicine papers [2], [3].

The nascent polypeptide is a 543 amino acid pre-proenzyme, which after removal of the signal peptide sequence in the endoplasmic reticulum, undergoes proteolytic processing in late endosomes/lysosomes by cathepsin-L like proteases [4] at sites Glu109-Ser110 and Gln157-Lys158, yielding a N-terminal 8 kDa polypeptide, a C-terminal 50 kDa polypeptide and between them a 6 kDa linker polypeptide [3]. The 50 and 8 kDa polypeptides associate to form a heterodimeric active enzyme, whilst the 6 kDa linker is excised and degraded [5], [6].

Heparanase activity is associated with activated leukocytes, mast cells, placental tissue and macrophages and the enzyme is secreted by activated CD4 + T cells [7], [8], [9], platelets [3], neutrophils and metastatic cells [10]. Upon secretion of heparanase from metastatic tumour cells, the enzyme hydrolyses the glycosidic bonds of heparan sulfate chains attached to proteoglycans to a product of 10–20 sugar units in length [11], leading to penetration of the endothelial cells of blood vessels and target organs by the tumor cell. Liberation of bound cytokines and growth factors sequestered by heparan sulfate chains in tissues [12] further facilitates growth of the tumour and promotes angiogenesis and proliferation of secondary tumours [13]. Levels of heparanase expression in tumour cells correlate with their metastatic potential; elevated levels of heparanase mRNA and protein have been found in cancer patients who show significantly shorter postoperative survival times than patients whose heparanase levels are normal [13], [14].

Heparanase upregulation in cancer cells from myeloma, lymphoblastoid and breast cancer reflects in augmentation of exosome secretion with an enhanced content of syndecan-1, VEGF and HGF whose roles are closely related to tumor aggressiveness [15]. In addition to its function in cancer progression, heparanase enzyme also plays a major role in inflammation *per se* and carcinogenesis related to inflammatory process [16]. The enzyme has been detected in a variety of immune cells including T and B cells, macrophages, neutrophils and mast cells. It has been shown to mediate extravasation through the endothelial barrier via the remodeling of ECM heparan sulfate, which then allows trafficking to the sites of inflammation [10], [17], [18]. Heparanase expression has been linked to tumorigenesis in a number of different cancers, for example, acute myeloid leukaemia [19], bladder, brain [20], breast [21], colon [22], gastric [23], oesophageal [24], oral [25], pancreatic [14], and cervical cancer [26], suggesting that it may be a suitable target for drug therapy. Currently available inhibitors of heparanase include neutralizing antibodies [27], peptides [28] and small molecules [29], [30].

A number of modified heparins and sulphated oligosaccharides have also been shown to be potent heparanase inhibitors with promising anti-tumour activities and have now advanced to the clinical testing stages. Examples of these include SST0001, M402, PI-88 and PG545. SST0001 is a fully N-acetylated modified heparin which lacks anti-coagulant activity and shown to be a selective heparanase inhibitor. It is currently in Phase I/II clinical trials for treatment of myeloma patients. M402 is an N-sulfated modified heparin that binds a wider range of growth factors compared to SST0001. This has progressed to Phase I/II clinical trials as a combination therapy with the chemotherapy agent gemcitabine for the treatment of metastatic pancreatic cancer. PI-88 is a sulphated polysaccharide with potent anti-angiogenic and anti-metastatic activity and with reduced unwanted anticoagulant activity. It has reached Phase III clinical trials for post-resection hepatocellular carcinoma. PG545 is a tetrasaccharide which has superior pharmacokinetic properties due to its high degree of lipophilicity. It has shown potent anti-tumour activity in PI88 resistant models, however, Phase I clinical trials in late 2010 were abandoned due to unexpected injection site reactions [31].

Aptamers are short DNA or RNA oligonucleotides developed for diagnostic and therapeutic use that display high binding affinity and specificity for target molecules [32]–[34]. The affinity of aptamers has been compared with that of antibodies (i.e. in the nanomolar range), but as aptamers are smaller (8–25 kDa compared to the 150 kDa size of antibodies), they can both penetrate tissues and be cleared from the plasma within minutes of intravenous administration without triggering an immune response, which can be useful when using them as diagnostic agents [35]. For therapeutic use, they are able to retain their function and binding characteristics upon modification with other moieties to improve their stability and solubility, whilst reducing their toxicity and plasma clearance [35]–[38] [39]–[41]. Typically, aptamers are from 22 to 100 bases in length, and contain a region of variable sequence, flanked by known sequences, which are used for amplification and identification purposes. A large repertoire of different sequence combinations (typically in the region of 10^15^) in the central domain creates many different folding arrangements, specificity and binding affinity for different molecules. Aptamers are typically produced based on the SELEX (systematic evolution of ligands by exponential enrichment) procedure [42], although a number of other selection methodologies are currently available [43]–[45].

Aptamers were previously generated against active human recombinant heparanase using a modified SELEX protocol and salt elution series. Selection yielded three aptamers, ‘1.5 M short’, a 30 base truncated version of ‘1.5 M long’ (73 bases), and ‘3.0 M’ (55 bases). ELISAs and fluorescence titrations separated the two longer aptamers as showing higher affinity and recognition of heparanase in placental cells, whereas placental tissue staining favoured ‘1.5 M long’. This was confirmed in a Matrigel invasion assay using ovarian carcinoma cells previously shown to require heparanase for invasion [46]. Two additional aptamers, termed ‘pink’ and ‘yellow’ were selected against the linker peptide sequence of pro-heparanase, as these could have a function in inhibiting the formation of the active heterodimer enzyme, by blocking peptide protease excision.

In this study, efforts were made to further characterise the previously selected aptamers and to assess their potential as a diagnostic or therapeutic agent. The stability of the aptamer was assessed by incubation over different time points with human and mouse serum, and polyacrylamide gel electrophoresis used to determine the extent of its degradation by nucleases present in the serum. An additional invasion assay, in addition to the previous mouse EHS-tumour derived Matrigel invasion assay, was carried out using human uterine leiomyoma tissue and heparanase-expressing human oral squamous carcinoma cells (HSC-3), as this experiment represents a more authentic picture of what happens in human tissue. Furthermore, a cell cytotoxicity/cell proliferation assay was performed to verify that any inhibition of invasion observed was not a result of cytotoxicity on the part of the aptamers. Finally, the interactions of the aptamers with serum proteins was investigated to both verify specificity of the aptamers and study their potential transport by such proteins in the bloodstream. Increasing literature in the DNA aptamer field has demonstrated that these molecules have tremendous therapeutic potential in cancer therapy treatment and have already been used as so called escort molecules to deliver drugs into cancer cells (reviewed in [47]). AS1411 is an example of a DNA aptamer that has progressed to clinical trials testing. The aptamer in this case specifically targets nucleolin protein and has been trialed with metastatic, clear-cell, renal cell carcinoma patients who have been refractory to prior tyrosine kinase inhibitors [48].

## Materials and Methods

### Cell culture

Human tongue squamous carcinoma cells, HSC-3 (JCRB 0623; Osaka National Institute of Health Sciences, Osaka, Japan), were cultured in growth media: 50% DMEM 50% Ham's F-12 (Sigma Aldrich) and additionally supplemented with 50 µg/ml ascorbic acid, 250 ng/ml amphotericin B, 5µg/ml insulin (bovine pancreas), 0.4 ng/ml hydrocortisone and 10% heat-inactivated foetal bovine serum. The culture supplement was purchased from Sigma Aldrich. All cell cultures were carried out using pre-warmed reagents. Cells were incubated in 95% air/5% CO_2_ at 37°C. Cells were passaged by removing media and washing with HBSS (Sigma Aldrich), then adding 3 ml 1× Trypsin-EDTA (Sigma Aldrich) and incubating for 5 minutes. The Trypsin-EDTA was inactivated by adding 7 ml growth media and removing any cell clumps. 1 ml cell suspension (plus 24 ml fresh growth media) was retained in the flask for maintenance of stocks and the remaining 9 ml was counted and used for experiments.

### Organotypic invasion assay and analyses of the inhibitors on invasion

The organotypic invasion assay and the quantitation of results were performed as described in Nurmenniemi *et al*. (2009) [49]. Briefly, 7 × 10^5^ HSC-3 cells suspended in media containing the appropriate aptamer (Unrelated, 1.5 M short, 1.5 M long, 3 M, Pink and Yellow) or an antibody against heparanase (Hpa Ab, 0.7 mM). Also (2-{4-[(E)-3-(4-bromophenyl) acryloylamino]-3-fluorophenyl} benzooxazol-5-yl) acetic acid, abbreviated to BAFB, was used, as this was shown to have inhibitory effects upon heparanase in previous studies [29] (Table 1). Each aptamer or antibody was added at 1 µM to HSC-3 cell suspension in the beginning of the study and in the HSC-3 cell culture media throughout the experiment. Myoma disks without HSC-3 cells and HSC-3 cells without inhibitor were also included in the assay as controls. The disks were incubated for 14 days at 37°C with 5% CO_2_ with the media containing the appropriate inhibitors. The media were collected, centrifuged, and fresh media with inhibitors were changed at days 4, 7, 10 and 14. From the collected media supernatants, the degradation products of myoma tissue type III collagen were analyzed using SP99 radioimmunoassay (RIA) for C-terminal telopeptide (IIICTP), and N-terminal telopeptide (IIINTP) indirect enzyme immunoassays (EIA) for N-terminal telopeptide, following the methods described in Nurmenniemi *et al*. ([49] for RIA and [50] for EIA). On day 14, the myoma disks were fixed in 4% paraformaldehyde and prepared for immunohistological analysis. Six µm histological sections of myoma disks were stained with monoclonal pancytokeratin antibody (DAKO, clone AE1/AE3 at a 1∶150 dilution) and viewed under a microscope at 100 × magnification. Nine representative images were taken from each of the three repeats of every treatment. Images were analyzed as described in Nurmenniemi *et al.* (2009) [49]. Differences in the invasion area and depth were evaluated using a Student's t-test and Mann-Whitney test and p-values less than 0.05 were considered statistically significant.

**Table 1:** The aptamer sequences used in this study.

Name	Sequence
1.5 M Long	GGGAGACAAGAATAAACGCTCAAATGG **ACTTTTGAATGTGGCAACAAATTCGACAGG** AGGCTCACAACAGGC
1.5 M Short	ACTTTTGAATGTGGCAACAAATTCGACAGG
Pink	TTGCTCCTTATAGAGCCGTCCGAGC
Yellow	CTAAAGTGCCTCACGCTGTTAACTC

In bold the sequence of the short aptamer, which is the part of the long aptamer that is structured.

### Cell proliferation assay

To determine the effect of the ‘1.5 M short’ aptamer on HSC-3 cell proliferation, we used the CellTiter 96 AQueous Cell Proliferation Assay (Promega), an MTS assay. Approximately 1 × 10^4^ cells were seeded in triplicate for in a 96-well plate with 1µM of the short aptamer. After 24, 48 and 72 h, 20 ml of CellTiter 96 Aqueous One Solution Reagent were added to each well and cells were incubated for 1 h at 37°C in a 5% CO_2_ incubator. The absorbance recorded at 490 nm on a FLUOstar Optima plate reader was used as a representation of the relative number of living cells in culture.

### Serum stability assay

Aptamers ‘1.5 M short’, ‘1.5 M long’ and ‘3.0 M’ were incubated at a concentration of 5 µM with human and mouse serum for 30, 60, 120, 180, 240 and 300 minutes at 37°C. The reaction was then stopped by the addition of 100 mM EDTA and the products ran on a 12% native polyacrylamide gel, alongside a 25 bp DNA marker ladder. Gels were stained using ethidium bromide and viewed under UV light.

### Serum albumin binding

Bovine Serum Albumin (BSA) was purchased from Sigma-Aldrich Ltd (Gillingham, UK, product code A7030 10 g). UV experiments were conducted on a Bio-Tek Uvikon XL with a Peltier Thermosystem for temperature control and stirring facility, connected to the PC utilizing Lab Power Junior software for data collection and analysis. Fluorimeter used was a Horiba Jobin Yvon Fluoromax-P equipped with a photon counter and Peltier system for temperature control and stirring facility, coupled to a PC utilizing Datamax software for spectral analysis. Initial measurements were taken to verify the presence or absence of fluorescent emission of both aptamers for excitation wavelength of 290 nm (selective for tryptophan residues) and emission wavelengths between 300 and 400 nm. Both aptamers were titrated in water and 10 mM phosphate buffer solutions pH 7.4, at 37°C. The 1.5 M short aptamer concentration varied from 0.3 to 8.0 µM, and the 1.5 M long aptamer varied from 0.5 to 8.0 µM, showing the intrinsic fluorescence of these aptamers. Both aptamers presented fluorescence emission spectra in this range. Earlier tests showed that aptamer concentrations ranging from 0.1 µg/ml to 8 µg/ml did not interfere in the evaluation of albumin quenching [51]. Quenching measurements were taken in 1 ml of 6 µM albumins in phosphate buffer pH 7.4. Emission spectra were registered from 300 to 400 nm wavelength, after a reaction time of 90 sec from each aptamer addition. Both emission and excitation bandwidth were set to 3 nm. Aptamer was added from a concentrated stock solution so that the volume increment was negligible. Experiments were performed at 37°C, pH 7.4.

To evaluate any existing primary and/or secondary inner filter effects (IFEs), correction procedures based on absorbance measurements of solutions were performed at excitation and emission wavelengths of albumin. This effect consists on the absorption of exciting and/or emitted radiation by dissolved species, including the fluorophore itself [52]. Absorbance measurement of aptamers/albumin solutions at excitation and emission wavelengths of albumin showed that inner filter effect caused by absorption of emitted radiation was negligible.

## Results

### The anti-heparanase aptamers inhibit carcinoma cell invasion

**Figure 1:**
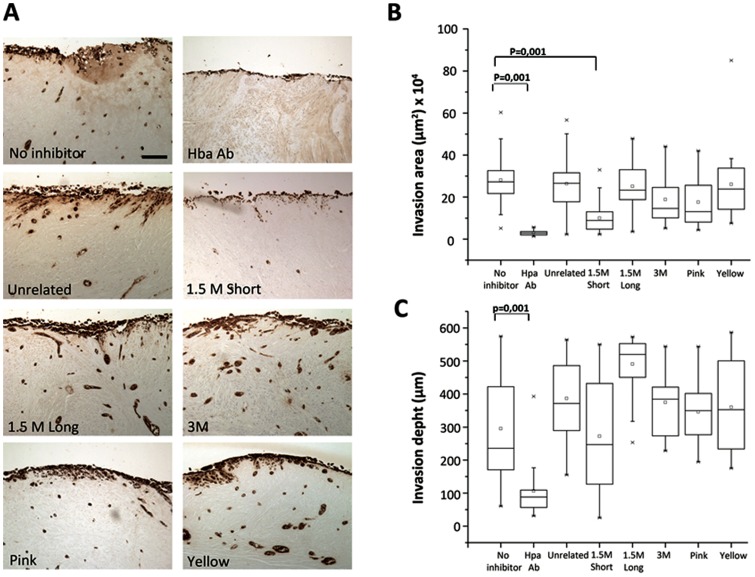
HSC-3 invasion in myoma discs. **A:** Paraffin-embedded 14-day myoma organotypic sections were stained for pancytokeratin marker AE1/AE3 to analyze HSC-3 invasion after various treatments: no inhibitor, Hpa Ab, (the polyclonal heparanase antibody as a positive control), unrelated aptamer, (selected against a target involved in Alzheimer's disease), anti-heparanase aptamers 1.5 M Short, 1.5 M long and 3 M; linker peptide aptamers Pink and Yellow. Scale bar is 100 µm. The differences in invasion area (B) and invasion depth (C) after various treatments (n = 27/treatment). The statistics were done as two-sample *t*-test and Mann-Whitney test.

**Figure 2:**
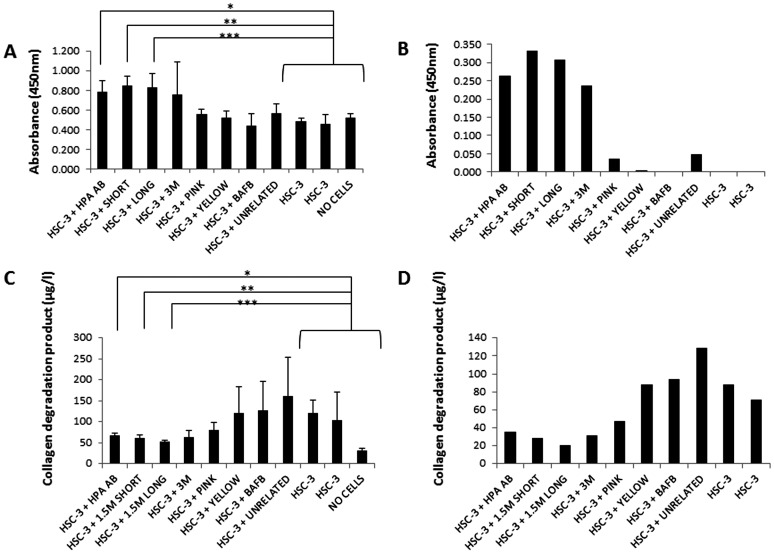
EIA and RIA assays. A: EIA (IIINTP indirect enzyme immunoassays) detecting N-terminal telopeptide from collagen type III degradation products at day 10 media change. Increasing absorbance means less collagen degradation product present. Hpa Ab (p = 0.03), 1.5 M Short (p = 0.007) and 1.5 M Long (p = 0.03) showed significant increase in absorbance compared to no inhibitor, suggesting they have inhibited the invasion of HSC-3 cells. B: The graph shows previous EIA values adjusted for negative control at day 10 media change. C: RIA (radioimmunoassay for type III collagen) detecting C-terminal telopeptide at day 10 media change. Increasing levels mean less collagen degradation product. Hpa Ab (p = 0.07), 1.5 M Short (p = 0.004) and 1.5 M Long (p = 0.02). D: RIA has confirmed the EIA assays showing significantly lower collagen degradation products than that for tissues without inhibitor added, indicating that they were successful inhibitors of invasion.

The invasion of HSC-3 cells was studied with a human myoma organotypic model [49] exposing the carcinoma cells to various aptamers (Unrelated, 1.5 M short, 1.5 M long, 3 M, Pink, and Yellow) or heparanase antibody (Hpa Ab) (Fig. 1A). The effects of these compounds compared to control (no inhibitor) on invasion area (calculated based on the µm-area of invasive cells) and depth of invasion (the distance from the lower surface of the noninvasive cell layer to the deepest invaded cell) were analyzed (Fig. 1B and C). Aptamer 1.5 M Short decreased significantly the total invasion area (p = 0.0001) similar to Hpa Ab, which was used as a positive invasion inhibitor control [27]. On the contrary, none of the other compounds had an effect on HSC-3 invasion (Figure 1). The invasion depth decreased significantly only after Hpa Ab treatment (p = 0.0001) (Figure 1C). Based on our previous findings, the degradation of type III collagen by HSC-3 cells measured with RIA peaks from days 7 to 11 [49]. Similarly, the media analyzed by RIA from day 4 did not show differences between any of the treatments (not shown). The media change upon termination of the experiment at day 14 showed only statistical significance from the treatment with no cells added (p = 0.001; not shown). Degradation of type III collagen without inhibitors was highest at days 7 (not shown) and 10 (Figure 2A–D). On day 10, the treatment with heparanase antibody inhibited significantly the degradation compared to unrelated control (p = 0.07 in RIA, and p = 0.03 in EIA). On day 10 there was a significant inhibition by 1.5 M Short (p = 0.004 in RIA, and p = 0.007 in EIA) and 1.5 M Long (p = 0.02 in RIA, and p =  0.03 in EIA) compared to HSC-3 control. However, 3 M, Pink, and Yellow aptamers, as well as BAFB showed no significant decrease in the amount of collagen degradation products, indicating that they were not successful inhibitors of invasion.

### The short anti-heparanase aptamer does not exhibit any cytotoxicity

**Figure 3:**
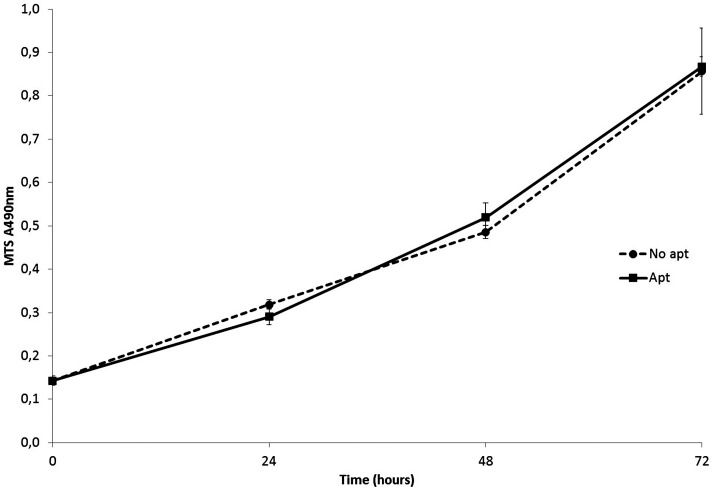
The MTS absorbance at 490 nm is shown over 24, 48 and 72 h in the presence and absence of the 1.5 M short aptamer. The presence of the aptamer at 1 mM concentration was found to have no effect on the cell growth in comparison with the control.

The cytotoxicity of the selected aptamers on HSC-3 cells was studied, to verify that the inhibition of invasion observed in the organotypic model was a result of the inhibition of the heparanase, as previously verified [46] and not cell cytotoxicity. The MTS assay was performed over 72 hrs, with a single addition of the aptamer in the beginning of the assay, and measurements over the period intervals of 24, 48 and 72 hrs. No change in cell viability and cell growth was observed between the cells where aptamer was added and the control (see Figure 3). Only the aptamers that showed inhibition of invasion were tested for cytotoxicity, to investigate if the inhibitory effect observed was due to cytotoxicity or inhibition of heparanase. Aptamers that do not inhibit cell invasion clearly have no effect on the cells and therefore there was no reason to be further studied for cytotoxicity.

### The short anti-heparanase aptamer does not bind significantly to serum proteins

**Figure 4:**
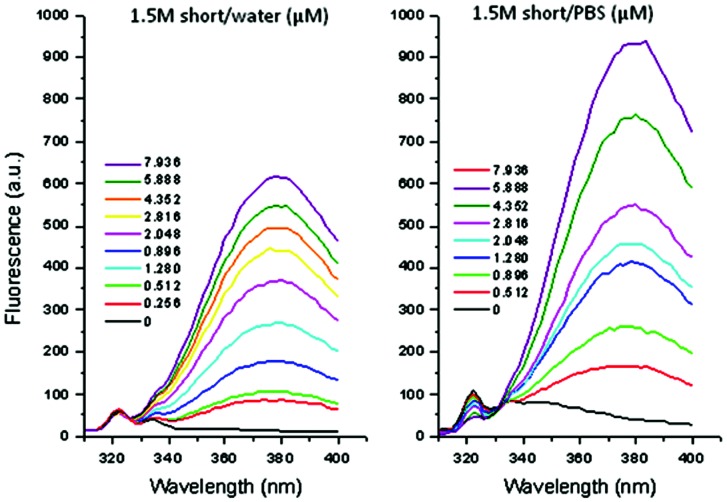
Fluorescence spectra of short aptamer in water and phosphate buffer solution (A) and PBS (B), at 37°C. Fluorescence increases upon increasing the concentration of aptamer in both phosphate and water, showing that although the fluorescence is higher in phosphate, the aptamer is in fact the cause and the pH difference in water and PBS is the most likely reason for the increase of fluorescence of the aptamer in PBS.

Short and long aptamers 1.5 M were initially titrated stepwise into water and phosphate buffer solution, pH 7.4 at 37°C to investigate their intrinsic fluorescence. Both aptamers have intrinsic fluorescence with peaks at 380 nm, which increases in fluorescence intensity upon a corresponding increase of the aptamer concentration; the short showing less fluorescence than the long aptamer (Figure 4A and B). Water was used as a diluent to show that the aptamer and not phosphate buffer solution was the cause of the fluorescence, although the fluorescence for the short aptamer increased upon using phosphate buffer solution as the diluent. However, this was likely due to a difference in pH as in fluorescence spectroscopy pH changes have a significant influence on results.

**Figure 5:**
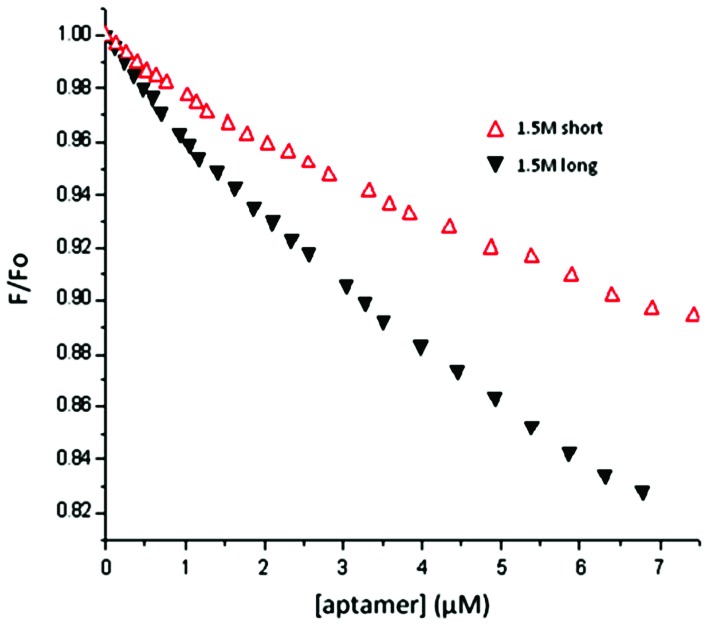
Stern-Volmer plots for HSA titrated by short and long apatmers 37°C. Excitation wavelength 290 nm, [HSA]  =  6 µM. Excitation wavelength at 290 mM in a solution of sodium phosphate. Data is the mean of six values showing no greater standard deviation than 11%. The quenching effect is more considerable for long than short aptamer.

The short aptamer was able to quench the fluorescence of HSA at 37°C by 1.5% (±0.03%) and 9% (±1.2%) at 1∶100 and 1∶10 molar ratios respectively, with quenching of 10% achieved by a molar concentration 9.1 times lower than HSA (Figure 5). Long aptamer is able to quench the fluorescence of HSA by 10% at a concentration 18.2 times lower than HSA, and by 2.4% (±0.1%) and 16% (±0.6%) at 1∶100 and 1∶10 molar ratios. The results show that both aptamers are able to quench the fluorescence of HSA, although the long aptamer was more effective. HSA quenching indicates that the aptamers reach sub domain IIA, where its single tryptophan is located. This tryptophan residue is located at site 214 in subdomain IIA, within which there is a large hydrophobic cavity with many arginine residues near the surface [53], which have been shown in different studies to serve as anchor points for aptamers [54].

To gain more information about the type of interaction occurring between the aptamers and HSA, UV spectrometry titrations were carried out by titrating increasing concentrations of short and long aptamers and 6 µM HSA diluted in phosphate buffer pH 7.4, as shown in Figure 5. The addition of both aptamers to phosphate buffer and HSA increased the overall absorbance, showing that the aptamer was responsible for this increase rather than HSA. The increase was more pronounced for the long aptamer over the short aptamer, and both produced a shift of the maximum absorbance to the left upon addition of increasing concentrations of aptamer.

**Figure 6:**
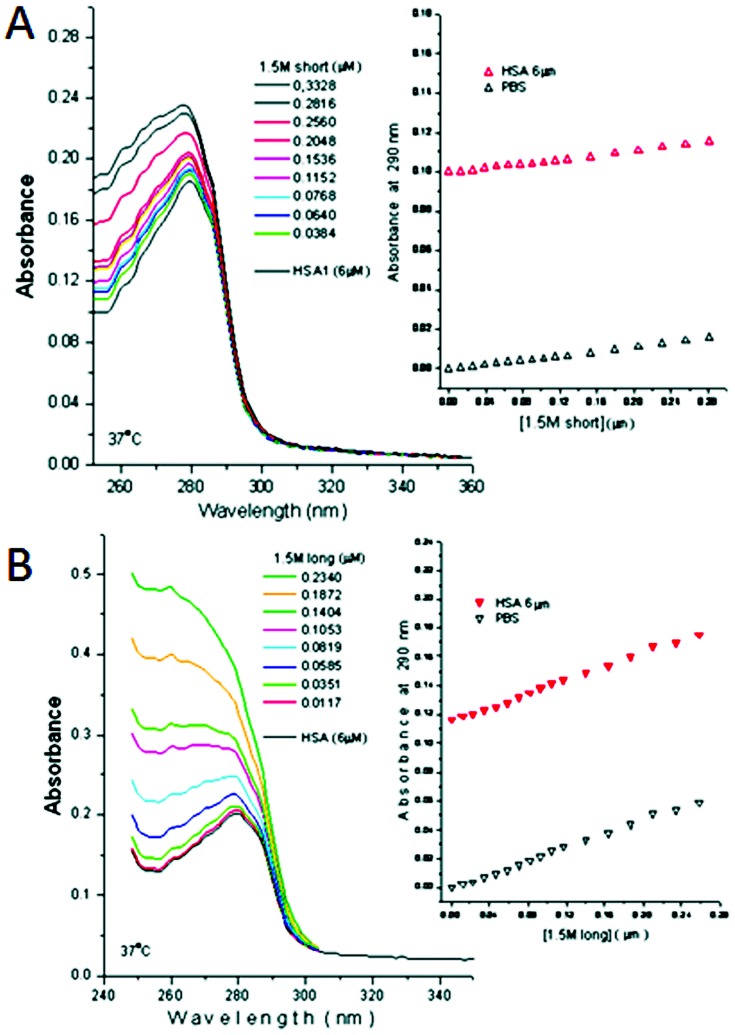
UV wavelength scan of HSA (left) and plot of PBS (right) titrated with 1.5 M short aptamer (A) and UV wavelength scan of HSA (left) and plot of PBS (right) titrated with 1.5 M long aptamer (B).

The shift observed from the short aptamer (Figure 6A) moved 6 nm to the left, suggesting that only a slight conformational change in the protein was occurring [55] and therefore, HSA quenching by this aptamer is most likely due to dynamic quenching. However, in the case of the long aptamer (Figure 6B), not only was there a substantial shift in the maximal absorption by 20 nm to the left, but a complete change in the shape of the peak was observed, incorporating the peak at 260 nm of the aptamer, suggesting that one complex was formed and that the quenching was due to the static quenching phenomenon with long aptamers [55]. Thus, the UV titrations suggested that the short aptamer did not form a complex with HSA and that the interactions were due to dynamic quenching, whereas the long aptamer was suggested to form a ground state complex with HSA, in contrast with other aptamers previously studied, which also show specificity and complex formation only with their target protein [56]. This work has been expanded and interactions of the aptamers with serum proteins and the specific position of their interaction has been calculated and published separately, as it was not within the scope of this article [57].

### Aptamers are stable in human serum

To assess the aptamers' suitability as therapeutic agents, it was necessary to have an understanding of how stable the unmodified aptamers would be in the body, as, in the bloodstream alone, there are many nucleases capable of degrading the aptamers. Thus, to verify the stability of the aptamers in human serum, we have characterized any degradation products by gel electrophoresis. Comparison of bands on the gels for 1.5 M short aptamer incubated for different time points with human and mouse serum, with that of aptamer only showed that 1.5 M short aptamer was not subject to nuclease degradation from human serum as the bands did not show any smearing or decrease in size or intensity compared to aptamer only, and hence, no breakdown of the aptamer into smaller fragments was observed (data not shown). With mouse serum, however, there was a decrease in primary band intensity at five hours' incubation time, suggesting that nucleases have degraded the aptamer by that time.

## Discussion

In this study we have explored the potential of previously selected aptamers against heparanase as promising diagnostic and therapeutic agents against oral cancer. The aptamers were previously shown to have high affinity against heparanase and were functional in a Matrigel assay. On these initial studies, it was found that the longer aptamers had a higher affinity for heparanase and they had performed well in fluorescent microscopy and Matrigel invasion assays. However, when we examined these aptamers on the organotypic invasion assay and analysed their potential to block invasion, it was found that the short aptamer was far more capable of doing so, compared to its long counterparts. This was also verified by the analysis by RIA and EIA of the degradation products of myoma tissue, namely type III collagen C- and N-terminal telopeptide respectively. The 1.5 M Short and 1.5 M Long aptamers consist of the same variable region and in fact the short one is a truncated version of the long. However, it appears that although the long one has a slightly higher affinity, probably due to increased interactions between the protein and the primer parts of the aptamer, these resulted in reducing the ability of these aptamers to inhibit tissue invasion. The presence of various proteins in the actual tissue, as compared to the Matrigel experiment previously performed, may be the reason for this, as the long aptamer may form other interactions with such proteins, or the primer tails may have a steric hindrance effect on the tissue, which is not apparent in the simpler matrigel model. This, in fact, was confirmed by the study of the interactions between the two aptamers and serum proteins. In these studies it was found that the long aptamer formed a complex with human serum albumin, whereas the short aptamer did not form a complex and showed only a limited dynamic quenching. In a further study [57], we have modelled the interactions of the short and long aptamers with HSA and have identified that indeed the long aptamer forms a complex with serum albumins in a single binding site, close to Trp 214 of HSA or 212 of BSA, at the subdomain IIA of these proteins, in a positively charged cavity lined with lysine and arginine residues [57]. It has been demonstrated that the shorter aptamer species lacks the ability to form complexes with serum proteins and exhibits thus higher specificity for its target, which justifies our choice of using it in any further therapeutic or diagnostic development and is in agreement with the myoma data presented in this work. One further important feature of this study is the demonstration that post-SELEX modifications may be more beneficial for aptamer selection than initial counter-selection steps, where this is possible. In a series of studies with various methodologies of detection, aptamer affinity for their target has been compared to that for albumin. The majority of the exemplars for new aptamer-based detection methodologies are based on the thrombin aptamers. In a study of aptamer-enhanced laser desorption/ionization study, the thrombin-binding DNA aptamer was used for affinity capture of thrombin in MALDI-TOF-MS. This aptamer was shown to be capable to bind to thrombin in a thrombin/albumin mixture [58]. Similarly, aptamers have been shown to distinguish thrombin from albumin in a QCM experiment [59]. Another G-quadruplex based thrombin aptamer in cationic polythiophene protein detection arrays was also able to detect thrombin over albumin in the attomole range in less than one hour without any tagging of the target [60]. The thrombin aptamer has also been used in an electrochemical detection assay, where it has been able to separate thrombin from BSA, HSA, Lysozyme and immunoglobulin G [61].

Apart from the thrombin aptamers, other aptamers in detection assays have also been compared with albumin, or have shown specific binding in the presence of high concentration of albumins. In an electrochemical sensor, aptamers against lysozyme have been shown to detect lysozyme in a mixture of six proteins including albumin [62]. Immunoglobulin E has also been detected in serum over albumin [63], whereas an anti- *F. tularensis* aptamer cocktail, when tested in a sandwich Aptamer-Linked Immobilized Sorbent Assay (ALISA) and dot blot analysis, exhibited specificity in its ability to bind only to tularemia bacterial antigen from subspecies *japonica, holarctica* (also known as *palaearctica*) and *tularensis* but not to *Bartonella henselae*, nor to pure chicken albumin or chicken lysozyme, demonstrating the ability of this aptamer cocktail to function as a bacterial detection agent [64].

Depending on the aptamer species, some aptamers present cross-reactivity with serum albumins, whereas the majority of them are capable of distinguishing between the protein they have been selected for, and albumins. Thus, for example, when we investigated a number of KLK6 aptamers with serum albumins, we identified that the majority of the selected aptamers against that target were specific, but one of them had significant affinity for albumin [65]. In addition, it is important to note that the same aptamer may or may not form complexes with HSA or BSA, depending on their post-SELEX refinement. Thus, the heparanase aptamer of this study, when truncated for the binding site of the specific target protein, does not form a stable complex with serum proteins, whereas its longer counterpart that contains the flanking primers, not selected for specific binding, can do. This is important with respect to selection strategies, as, in the case of heparanase, we started the selection with a naïve library, containing all possible species, and the selection and studies clearly indicated that one aptamer species was the best candidate. This candidate would have been lost if a counter-selection against albumins had been performed at the beginning, as it also presents affinity for these proteins prior to truncation. However, with a simple truncation of the flanking primers, the aptamer gained the necessary specificity to be further developed for therapeutic and diagnostic applications.

Furthermore, the effect seen in the inhibition of the invasion assay could have been a result of a cytotoxic effect on the part of aptamers. This possibility was eliminated in a cytotoxicity assay, which clearly demonstrated that the aptamers did not show any cytotoxic effect on these cells after 72 hours of incubation, thus verifying that the inhibition of invasion was in fact due to inhibition of heparanase. Finally, aptamers were found to be stable in human serum even without any modification, making them potentially interesting therapeutic reagents on their own accord. This is important as such stability would reduce production costs of such an aptamer, if it were selected for subsequent therapeutic or diagnostic applications.
